# Optometrist to Operation: Patients’ Perspective on the Innovative Use of Quick Response (QR) Code-Linked Patient Information Video on Cataract Surgery

**DOI:** 10.7759/cureus.49336

**Published:** 2023-11-24

**Authors:** Wei Han Ong, Joanna Ashby, John Ellis

**Affiliations:** 1 Department of Ophthalmology, Ninewells Hospital, Dundee, GBR; 2 Department of Ophthalmology, Wolfson Medical School, University of Glasgow, Glasgow, GBR

**Keywords:** qr code, surgery, patient education, patient information, cataract

## Abstract

Background

This study aimed to explore patients’ subjective utility for a patient information video (PIV) on cataract surgery and analyse the use of a quick response (QR) code as a mode of delivery of the PIV.

Methods

A total of 500 patients were included in the study. All patients were given a paper form of the patient information leaflet (PIL) as the standard of care (SoC) in addition to a digital QR code to access a supplementary PIV. The questionnaire explored the patients’ understanding of cataracts, the risks and benefits of cataract surgery, and their experience accessing and using the QR code.

Results

A total of 321 responses were collected (64% response rate). The majority were female (55%), with a mean age of 75 years. Among these, 69% (n = 222/321) managed to watch the video. A statistically significant association was reported between prior experience with QR codes and the ability to watch the video (p<0.001). The most common reason for not watching the video was no device (n=54/99, 54%). Ninety-one percent of the patients who watched the video expressed a desire for additional healthcare videos in the future.

Overall, most patients (n=170/222, 76%) acknowledged that the PIV was easier to understand when compared to paper-format information, with a minority of patients reporting the PIV missing information that was covered on paper (n=2/222).

Conclusions

The provision of PIV supplementation as a part of the cataract surgery referral pathway is an innovative method of providing patient information in a more interactive way, with positive feedback from patients.

## Introduction

Digital health is having a profound effect on our healthcare systems, enabling new models of care, improving quality, efficiency, and patient experience, as well as supporting more integrated care and improving the health of the population. Cataract surgery is the most frequently performed surgery in the UK, with approximately 500,000 surgeries performed annually in England and Wales [1]. Although highly successful in technical and perceived value terms, no surgery is risk-free, and all elective surgeries require comprehensive, informed consent. This enables shared decision-making. We piloted an innovative approach to proposing a service adaptation to the cataract referral pathway locally by streamlining direct-optometrist referral to the listing for cataract operation with the use of patient information video (PIV) accessed via a quick response (QR) code.

## Materials and methods

This single-centre, prospective study was conducted at the ophthalmology department of Ninewells Hospital, Dundee, Scotland. A total of 500 patients with a diagnosis of cataracts made by a community optometrist were enrolled. These referrals included those with other sight involving ophthalmic pathologies (for example, degrees of dry or stable age-related macular degeneration), but the referral required the examining optometrist to commit to the conclusion that cataract was the principal reversible factor impacting vision.

**Figure 1:**
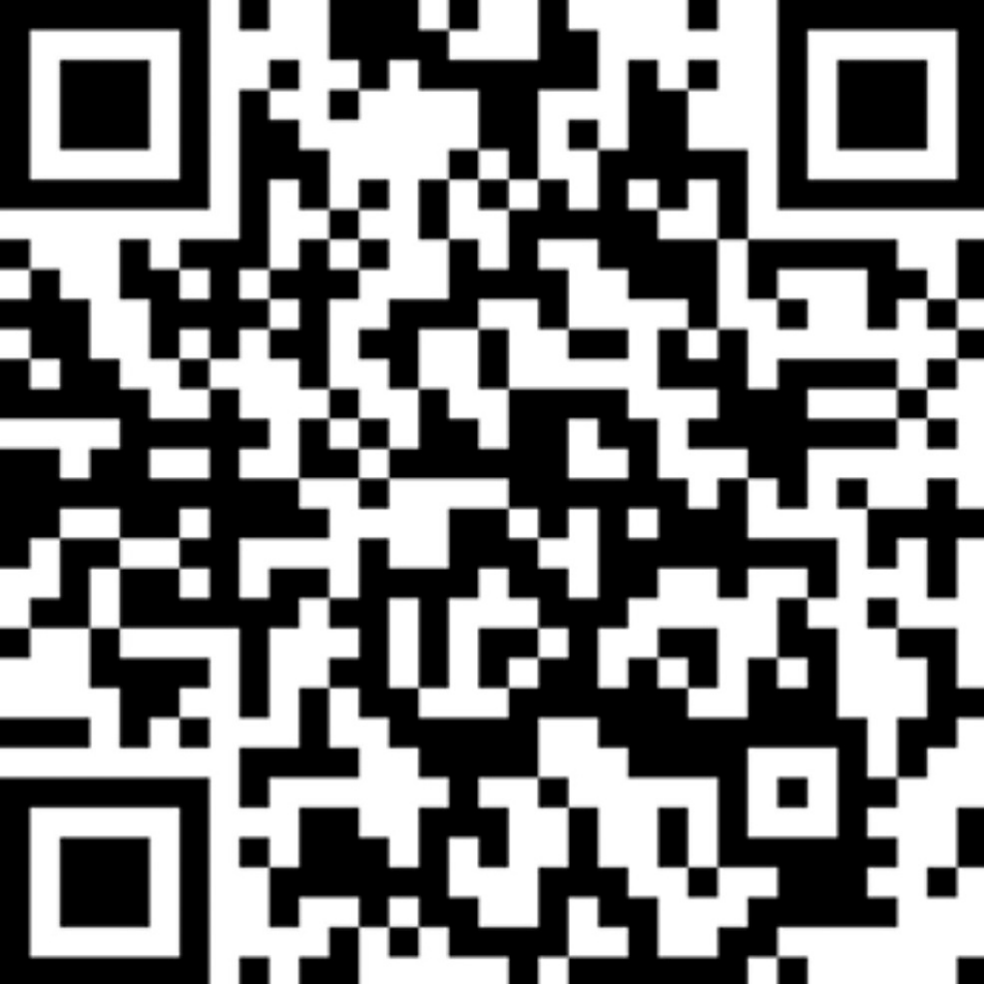
QR code for the video on cataract surgery QR: quick response

All patients were provided with a cataract letter pack, which informed the patients that they could now benefit from cataract surgery while highlighting that the surgery is voluntary, and the information enclosed aimed to help them make the correct decision regarding risk and benefit. The pack contained: 1) a cataract surgery consent form; 2) a cataract surgery information leaflet in paper form as the current standard of care (SoC); 3) a printed QR code linked to a supplementary patient information video on cataract surgery (Figure 1); and 4) a questionnaire that did not include the name or identifiable information.

Patients were given three options: to join the cataract operation waiting list, to decline, or to request a further phone call for more information. Questionnaires were then returned in prepaid envelopes directed to the local ophthalmology department.

The cataract surgery patient information video was produced with the involvement of the wider cataract team at Dundee Ophthalmology Department, Scotland, providing patients with unrestricted access to a four-minute, 30-second video showing the consent dialogue and all relevant aspects of the patient journey. No technical details of surgery or images that might be deemed upsetting were included. The video afforded the opportunity to interview other members of the team (theatre and ward nurses) as well as show the physical environment of the ward and theatre to render these more familiar when encountered. The video was titled “Your Cataract Surgery: A Patient Information Video” (Account: EYE Surgeon) and was made available as a character string (https://www.youtube.com/watch?v=JPaHjL8LUzs) and a QR code. The video was uploaded to YouTube as a public online video. The video is exclusively accessible in the English language, supplemented with closed captioning that encompasses crucial information. This video begins by discussing the diagnosis of cataract and its symptoms. It then explains cataract surgery in broad conceptual terms, followed by some pointers to risk and benefit, and refers to the standard printed sheets where more detail can be found. The end of the video describes what to expect after the operation, including the visual outcome, post-operative care, and follow-up. This video was made to cover key information for cataract surgery, similar to that in the patient information leaflet.

The questionnaire design consisted of two parts, with the first part aiming to address patient demographics, prior experience with QR codes, and the ability to access and watch the video. Patients who managed to watch the video were then directed to complete the second part of the questionnaire (see questions below), which used a five-point Likert scale to grade their knowledge of cataract surgery, satisfaction with the quality of the video, and preference for the format of information delivery. The following questions were rated as follows: strongly disagree, disagree, neither agree nor disagree, agree, strongly agree

• I found the video clear and easy to understand.

• I feel the information was too detailed.

• I feel I understood my treatment options.

• I feel I understand the risks, and I want to.

• I feel I was free to choose not to go ahead with surgery if I didn’t want to.

• I found the video easier to understand than the paper information.

• I found the video missed out on some important information the paper covered.

Other question types involved multiple-choice and full-text answers.

## Results

A total of 321 respondents completed the questionnaire (64% response rate). The age range of the respondents was 45-97 years (mean age: 75 years). Overall, 53% of respondents were aged over 75 years (approximately the mean age for cataract surgery in Scotland). The female-to-male ratio was 1.2:1. Overall, 69% of the cohort managed to watch the video (n=222/321). Of 321 participants, 42% (n=134/321) had previously used QR codes, presumably becoming more ubiquitous in restaurants and advertisements in the COVID era. Smartphones were the most widely used device (69%, n=154/222), and only 28% (63/222) of the participants used a tablet to watch the video. The owners of the device used were mainly the patients themselves (n=157/222, 71%), followed by family members (n=54/222, 24%), and less commonly, a device belonged to a friend (n=11/222, 5%). In terms of video viewing, most patients reported watching the video between one and two times (n=186/222, 84%), with a few repeating it three times or more (n=17/222, 8%). The most common reasons for not watching the video were no devices (n=54/99, 54%) and no internet access (n=41/99, 41%). Other recorded reasons included ‘not sure how to do this’ (26%) and ‘not interested’ (7%).

**Figure 2:**
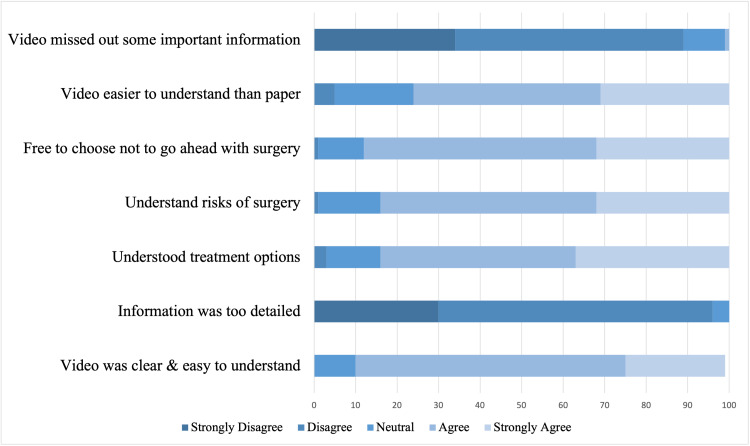
Patient's perspective on PIV using the five-point Likert scale PIV: patient information video

A five-point Likert scale was used to grade patients' understanding of the cataract surgery video (Figure 2).

The majority, 89% (n=199/222), of patients agreed or strongly agreed that the video was clear and easy to understand. In terms of the usefulness of the video in guiding decision-making for surgery, 84% (n=187/222) reported they understood the treatment options, while 83% (n=185/222) agreed or strongly agreed they understood the risks and benefits of cataract surgery as well as they wanted to. Significantly, 87% (n=194/222) agreed that they felt free to choose not to have surgery if they did not want to, implying the video or the large information pack did not result in pressure to ‘conform’. Generally, most patients (n=170/222, 76%) acknowledged that the PIV was easier to understand when compared with standard paper format documentation, with only two patients (n=2/222) reporting the video as missing information better or more fully covered by the printed information. Ninety-one percent of patients who managed to watch the video stated they would like to have more healthcare videos in the future.

The positive impact of previous QR code usage and the ability to watch the patient information video was found to be statistically significant in our study (p<0.001). With respect to age, increasing age was found to be negatively correlated with prior usage of QR codes (p=0.04) and predictive of an inability to watch the video (p=0.01). However, patients’ preference to have more healthcare information videos in the future was not significantly related to age or gender.

## Discussion

Ideally, the patient and doctor should have a personalised discussion regarding the procedure, benefits, risks, alternatives, and expected outcomes of all planned surgeries. Communication should be honest, the level of detail appropriate, and all exchanges should be open and free. It should be comprehensible and tailored. It should afford the opportunity for questions, and when all outcomes are considered, the option of doing nothing (no surgery) must always be explicit. Cataract surgery is clearly an exemplar for such ethically fair shared decision-making, as there is no biological urgency and the intervention is not time-bound or prejudiced by delay, within reasonable bounds. Several studies have shown that clear and effective patient education before surgery is imperative for increasing patient satisfaction and reducing pre-operative anxiety and post-operative complications [2]. This can be a challenge in the context of increasingly overburdened services, and few are more clearly so pressed as the ophthalmic surgical services, with demand for cataract surgery expected to increase by 25% in the next 10 years [1]. This is on the background of astonishing service increases historically and reducing human resources in healthcare [3].

Existing demands on productivity limit surgery and patient contact time available, which directly impacts the time available for appropriate consenting quality and the time available for the patient. This has been described as the efficiency-thoroughness trade-off (ETTO) [4]. This often results in the patient receiving an overwhelming amount of information in a short span of time during the stressful period of a new diagnosis and first consultation. Important relatives may be absent, and the patient may have hearing or other forms of sensory or cognitive impairment. Rendering the decision more complicated still, this is a highly emotionally charged time as patients are often anxious about the diagnosis due to the uncertainty associated with treatment options and outcomes, which may negatively impact the quality of consent and compromise patient information processing. Additionally, studies have shown that information retention regarding common ophthalmic surgeries generally ranges from approximately 40% to 70%, suggesting that the informed consent process may benefit from further improvements [5]. The use of patient information leaflets (PILs) is widely promoted by the General Medical Council (GMC) to encourage better patient participation in their healthcare. While the primary medium used for the provision of PILs remains paper-based, the possible benefits of using video-based media in the informed consent process have been gaining traction.

Video-based media are one of the most ubiquitous forms of communication in the modern age, as they provide a consistent form of educational information and communicate concepts realistically and visually. In ophthalmology, the effects of different presentation methods on patient knowledge have most commonly been investigated within the informed consent process for cataract surgery [6]. However, there is limited literature exploring the benefits of using QR code-enabled online video consenting. In this study, we take the innovative approach of proposing a service adaptation for the cataract referral pathway locally by streamlining direct-optometrist referral and listing to cataract operation with the use of a PIV through a QR code. To our knowledge, most studies to date examine the efficacy of digital healthcare services in enhancing clinical outcomes without focusing on patient engagement in their specificity. Our study aimed to explore the patients’ perspective on the use of the PIV to enhance the consent process for cataract operations and, subordinately, to analyse the ease of use of a QR code as a mode of delivery of that PIV, being conscious of the potential for digital inequity or other forms of the discriminative disutility of this chosen medium and form of delivery.

This Quality Improvement Project (QIP) took the form of a non-inferiority pilot in the sense that the innovation was compared with the current standard of care. It is obvious that with respect to normal consent, as practised in the National Health Service, there is no standardisation of what content is delivered, at what pace, and with what degree of detail and time for dialogue is admitted. One advantage of the PIV is that it can be so recorded as to ensure every person watching receives the same content and emphasis when these are agreed upon as the minimum ‘data set’ for any patient. One interesting and unanticipated finding was that, while only 69% of the cohort reported accessing the video, 71% (respondents) requested that they be placed directly on the waiting list for surgery. It is not obvious that the numbers overlap entirely, but certainly, some patients who opted for surgery had not viewed the PIV. Given that they were empowered and happy to do so, this may reflect that for many respondents, the printed information is sufficient. It must always be appreciated that if a patient declines any given degree of detail in an offered explanation in any consent process, that is one's right. As has been said, “A patient is using their autonomy when they choose to surrender their autonomy” [7].

The limitations of the current study are the inability to correlate surgical outcomes or Patient-Related Outcome Measures (PROMs) with the choice made and the predictive validity of the levels of confidence and satisfaction with the PIV and QR code delivery modality with acuity or quality of life outcomes. This is a limitation of the anonymous nature of the questionnaires used in this QIP. Furthermore, the paper-based delivery of the code to access the PIV means that recipients who do not possess an internet connection or a device cannot proceed. If the information is provided on a web-based programme or device on the optometry premises at the point of referral, access would be afforded by the definition of that modality. Such systems exist, and we propose to audit this next iteration of the PIV.

## Conclusions

Direct optometrist-listing for cataract surgeries (“Optometrist to Operation”) through a streamlined pathway incorporating video and QR code approach benefits patient satisfaction, autonomy, beneficence, and fairness while streamlining care delivery processes within the cataract team. The fulfilment of cataract surgery provision remains a continuous challenge within NHS Scotland. We show that with minimal investment, collaborative working with primary care services, and a smart redesign process, local provision and access can be possible.

## References

[1] The Royal College of Ophthalmologists Changes in NHS Cataract Surgery 2016-2021: an analysis of national, regional and independent sector trends. Changes in NHS Cataract Surgery in England 2016-2021: An Analysis of National, Regional and Independent Sector Trends. August 2022.

[2] Brodersen F, Wagner J, Uzunoglu FG, Petersen-Ewert C (2023). Impact of preoperative patient education on postoperative recovery in abdominal surgery: a systematic review. World J Surg.

[3] Ham C (2020). The challenges facing the NHS in England in 2021. BMJ.

[4] Hollnagel E (2012). The ETTO Principle: Efficiency-Thoroughness Trade-Off. Why Things That Go Right Sometimes Go Wrong.

[5] Kessels RP (2003). Patients' memory for medical information. J R Soc Med.

[6] Karan A, Somasundaram P, Michael H, Shayegani A, Mayer H (2014). The effect of multimedia interventions on the informed consent process for cataract surgery in rural South India. Indian J Ophthalmol.

[7] Entwistle VA, Carter SM, Cribb A, McCaffery K (2010). Supporting patient autonomy: the importance of clinician-patient relationships. J Gen Intern Med.

